# Effectiveness and safety of antiresorptive drugs in patients with systemic sclerosis—a retrospective monocentric study

**DOI:** 10.1016/j.ero.2025.05.008

**Published:** 2025-06-26

**Authors:** Nils Schulz, Jonas Neumann, Tim Wilhelmi, Pascal van Wijnen, Ulf Müller-Ladner, Philipp Klemm

**Affiliations:** Department of Rheumatology, Clinical Immunology, Osteology and Physical Medicine, Justus Liebig University Giessen, Campus Kerckhoff, Bad Nauheim, Hesse, Germany

## Abstract

**Objectives:**

Systemic sclerosis (SSc) is associated with an increased risk of osteoporosis. Gastrointestinal manifestations of SSc such as dysphagia and chronic gastritis affect up to 90% of patients and thus may impair the efficacy and safety of oral antiresorptive treatments. However, data on the comparative effectiveness and safety of antiresorptive therapies in SSc are lacking.

**Methods:**

Patients with SSc treated for osteoporosis at our rheumatology department between 2004 and 2024 were included if they had at least 2 dual-energy X-ray absorptiometry measurements 2 years apart (before and during antiresorptive therapy). Primary endpoint was the change in bone mineral density (BMD). Secondary endpoints assessed changes in BMD for individual drugs, with a specific focus on oral compared with parenterally administered drugs. Adverse events were documented descriptively.

**Results:**

A total of 42 patients were included. Overall, antiresorptive therapy significantly improved axial BMD (+3.78%, *p* = .012), whereas femoral BMD remained unchanged. Subgroup analysis showed a significant axial BMD increase with parenteral therapy (+5.46%, *p* = .026, n = 21), but not with oral drugs (n = 21). Denosumab yielded the highest axial BMD gain (+7.51%). Overall, no new fractures occurred. One patient developed stage 1 osteonecrosis of the jaw (ONJ) under ibandronate. Three of 19 patients (16%) developed mild gastrointestinal symptoms. No severe gastrointestinal symptoms occurred.

**Conclusions:**

Antiresorptive therapies appear to be effective in patients with SSc. Subgroup analysis suggests that parenteral administration, particularly denosumab, may offer superior efficacy. Gastrointestinal adverse effects were mild and consistent with published rates in the general population, whereas ONJ could be more relevant than in the general population.

**WHAT IS ALREADY KNOWN ON THIS TOPIC**

• Systemic sclerosis (SSc) patients are at increased risk for osteoporosis and fractures, partly due to systemic inflammation, vascular damage, and disease-related factors.
• Gastrointestinal involvement is common in SSc and may impair the efficacy and tolerability of oral antiresorptive osteoporosis therapies.

**WHAT THIS STUDY ADDS**

• This study provides new data on the effectiveness and safety of antiresorptive osteoporosis treatments specifically in patients with SSc, a group for which evidence has previously been very limited.
• It shows that parenteral antiresorptive therapies, particularly denosumab, lead to significant improvements in axial bone mineral density, whereas oral therapies demonstrate less pronounced benefits.

**HOW THIS STUDY MIGHT AFFECT RESEARCH, PRACTICE, OR POLICY**

• Suggests prioritizing parenteral antiresorptive agents in SSc patients to optimize efficacy and reduce gastrointestinal complications.
• Emphasizes the need for careful dental evaluation and monitoring for ONJ risk in this patient population.
• Highlights the importance of larger prospective studies incorporating comprehensive adverse event monitoring, adherence assessment, and bone metabolism markers to better guide osteoporosis management in SSc.

Alt-text: Unlabelled box

## INTRODUCTION

Systemic sclerosis (SSc) is an inflammatory rheumatic connective tissue disease characterised by abnormal autoantibody production and immune activation, vasculopathy, and excessive fibrosis [1]. Meta-analyses from recent years have shown that patients with SSc have a significantly increased prevalence of reduced bone mineral density (BMD), making them a particularly osteovulnerable population [[2], [3], [4]]. Contributing factors include demographic aspects such as older age and menopause, as well as disease-related factors such as longer disease duration, glucocorticoid exposure, and the presence of anti-RNA polymerase III antibodies [4,5]. In accordance with reduced BMD, patients with SSc experience a markedly elevated fracture incidence (reported in some cohorts to reach up to 38%) with an odds ratio of 10.38 for vertebral fractures [6]. Besides the considerable economic burden, major osteoporotic fractures are associated with substantial pain, immobility, and increased mortality [[7], [8], [9], [10]].

Gastrointestinal involvement, including dysphagia, gastroparesis, and malabsorption, affects more than 90% of SSc patients and can significantly impact quality of life [11,12]. Importantly, altered gastrointestinal motility may also interfere with the effectiveness and safety of oral osteoporosis treatments [13]. Oral bisphosphonates are a well-established therapeutic option in osteoporosis management and can significantly reduce fracture rates [14]. However, their oral bioavailability is limited to 1% to 6% and may be further reduced in the presence of malabsorption or delayed gastric emptying, such as in SSc-related gastroparesis. Prolonged contact with the upper gastrointestinal mucosa also increases the risk of ulcer formation [13,15].

The aim of this retrospective study (DRKS00033393) was to assess the efficacy and safety of oral compared with parenteral antiresorptive therapy in patients with SSc—a disease frequently associated with gastrointestinal involvement.

## METHODS

### Study design

The study is a retrospective evaluation of the effectiveness and safety of antiresorptive drugs in patients with SSc and treatment-requiring osteoporosis.

### Setting

Recruitment and data collection were conducted between February 7, 2024, and December 31, 2024, at the Justus Liebig University Giessen, Campus Kerckhoff in Bad Nauheim, Germany.

Data collection was performed through secondary analysis of pre-existing data obtained during routine clinical care as part of the diagnostic work-up and follow-up of our SSc cohort. Data were extracted from the hospital information system and the picture archiving and communication system.

The study was approved by the Ethics Committee of Justus Liebig University Giessen (AZ 158/23) on November 2, 2023, and prospectively registered in the German Registry of Clinical Studies (DRKS) under the number DRKS00033393.

### Participants

Patients with SSc who were treated at the Kerckhoff Clinic (Justus Liebig University Giessen, Campus Kerckhoff) in Bad Nauheim between 2010 and 2023 were included in the evaluation. They also had to meet the following inclusion and exclusion criteria:

#### Inclusion criteria

• 18 years of age
• Confirmed diagnosis of SSc that meets the American College of Rheumatology /European Alliance of Associations for Rheumatology classification criteria from 2013 [16]
• Administration of an antiresorptive therapy
• At least 2 BMD measurements taken 2 years ± 3 months apart (before antiresorptive therapy and during ongoing/completed therapy of 2 years)

#### Exclusion criteria

• Interrupted therapy with antiresorptive medication
• Osteoanabolic therapy
• Other secondary osteoporosis:
  • Glucocorticoids >5 mg prednisone equivalent/day
  • Type I diabetes mellitus
  • Therapies with growth hormones, estrogens, aromatase inhibitors, or other cancer therapies
  • Heart failure with a left ventricular ejection fraction <35%
  • Hyperthyroidism (except for factitious hyperthyroidism)
  • Chronic kidney disease Kidney Disease: Improving Global Outcomes stage G3b (Glomerular Filtration Rate <45 mL/min/1.73 m²)
  • Hyperparathyroidism

### Outcomes

Primary and secondary outcomes were assessed before the first and during the second BMD measurement.

### Primary outcome

#### BMD

Changes in axial and femoral BMD (in mg/cm² as well as T-score) in the overall cohort.

BMD measurements were performed using dual-energy X-ray absorptiometry with a Lunar Prodigy X device (Lunar Radiation Corporation). Measurements were taken at the lumbar spine (L1-L4) and the right femoral neck. According to WHO criteria, a decrease in BMD of >2.5 SDs below the mean value for young healthy adults (T-score < −2.5) was defined as osteoporosis. A T-score between −2.5 and −1 was classified as osteopenia, whereas a T-score > −1 indicated age-appropriate BMD [17].

### Secondary outcomes

#### BMD

Changes in axial and femoral BMD (in mg/cm² as well as T-score) depending on the route of administration (oral vs parenteral) as well as for the individual drugs.

#### Treatment failure

Defined criteria for pharmacological treatment failure in osteoporosis are not firmly established. According to the German Osteology Society (Dachverband Osteologie e.V., DVO) guidelines, treatment failure should be considered in cases of a significant decline in BMD (>5%) and/or the occurrence of 2 or more osteoporotic fractures within a 3-year period despite ongoing therapy [18].

#### Treatment response

Investigation of the dependence of treatment response on demographic data such as sex (female/male), age, BMI, as well as disease-associated contextual factors such as disease duration, form of SSc (limited vs diffuse cutaneous), antibody status (anti-centromere-antibody, anti-topoisomerase I-antibody, anti-RNA-polymerase III-antibody), organ manifestation (skin, lungs, vessels, oesophagus), C-reactive protein (CRP), vitamin D status, rheumatological medication, prednisolone therapy, proton pump inhibitor therapy, and antidepressant therapy.

The extent of any potential skin manifestation of the SSc was assessed using the modified Rodnan skin score [19]. This is used for the quantitative assessment of skin sclerosis and consists of evaluating skin thickness in 17 different body regions. Each area is rated on a scale from 0 to 3, where 0 represents normal skin and 3 indicates pronounced skin sclerosis. The points from all assessed regions are summed to obtain a total score, which ranges from 0 (no skin involvement) to 51 (maximum skin involvement).

A lung involvement was differentiated into the presence of an interstitial lung disease, which was diagnosed by a radiologist using high-resolution computed tomography (HRCT), and pulmonary arterial hypertension, which was diagnosed or ruled out by right heart catheterisation when there was sufficient suspicion.

The presence of digital ulcers was clinically defined by an experienced rheumatologist.

An oesophageal hypomotility or atony as a surrogate parameter for a manifestation of the upper gastrointestinal tract was diagnosed or excluded by a radiologist using HRCT.

#### Laboratory parameters

CRP (reference range, up to 0.5 mg/dL), serum calcium (reference range, 2.09-2.54 mmol/L), and 25-OH vitamin D (reference range, 20-70 ng/mL) were tested in the blood. To exclude potential confounding factors affecting bone metabolism (such as secondary osteoporosis), thyroid-stimulating hormone (reference range, 0.27-4.2 µU/mL) and serum creatinine (reference range, 0.5-0.9 mg/dL) were also measured.

#### Adverse events

Adverse and severe adverse events (the occurrence of atypical femur fractures, osteonecrosis of the jaw (ONJ), acute-phase reaction after infusion of zoledronic acid, hypocalcaemia, acute kidney failure, atrial fibrillation, heartburn, stomach pain, gastrointestinal ulcers, gastrointestinal bleeding) were documented descriptively.

### Statistical analysis

#### Descriptive analysis

Continuous variables were summarised using means and SDs. Changes between baseline and follow-up (Δ-mean) were calculated for each variable. Normality of distributions was tested using the Shapiro–Wilk test to determine the use of parametric or non-parametric tests.

#### Comparative tests

For comparisons within groups (eg, baseline vs follow-up BMD), the Wilcoxon signed-rank test was applied. For comparisons between 2 independent groups (eg, oral vs parenteral administration), the Mann–Whitney U test was used. To compare more than 2 treatment groups (eg, denosumab vs zoledronate vs alendronate), the Kruskal–Wallis test was applied. For categorical variables, the Chi-square test was used.

To explore potential predictors of treatment response, a multivariate linear regression analysis was conducted.

#### Significance level

A *P*-value <.05 was considered statistically significant. To adjust for multiple comparisons, the Bonferroni–Holm correction was applied where appropriate.

### Software and blinding

Statistical analyses were performed using R version 4.4.1 for Windows.

## RESULTS

### Inclusion and patient characteristics

Out of 194 screened patients with SSc, 129 (66%) had reduced BMD, including 64 (33%) with osteopenia and 65 (34%) with osteoporosis. Forty-two patients met the inclusion criteria (baseline characteristics; Table 1). The most common reasons for exclusion were incomplete data and failure to meet the treatment threshold for antiresorptive therapy. Antiresorptive therapies included alendronate (n = 19), zoledronate (n = 9), denosumab (n = 11), risedronate (n = 2), and ibandronate (n = 1). Thirty-one of 42 patients (74%) showed morphologically detectable oesophageal hypomotility or atony on CT, which was comparably distributed between treatment groups (oral: n = 14/21 [67%]; parenteral: n = 17/21 [81%]).

**Table 1:** Patient characteristics

Characteristic	n (%)/mean ± SD
Number of patients	42 (100%)
Demographic features:	
Female	41 (98%)
Age (y)	64.7 ± 9.6
BMI (kg/m²)	23.5 ± 4.9
Disease specific features:	
Disease duration (y)	11 ± 9
lcSSc	36 (83%)
dcSSc	6 (17%)
Autoantibody:	
- Anti-centromere	32
- Anti-topoisomerase I	9
- RNA Polymerase III	1
Organ involvement:	
mRSS	9.6 ± 8.3
Oesophageal hypomotility or atony (CT)	31 (74%)
DU	24 (57%)
ILD	22 (52%)
PAH	9 (21%)
Patients with osteoporotic fractures	7 (17%)
Laboratory values:	
CRP (mg/dL)	0.47 ± 0.58
Serum calcium (mmol/L)	2.35 ± 0.10
Vit D3 (ng/mL)	35.52 ± 12.82
Creatinine (mg/dL)	0.70 ± 0.12
TSH (µU/mL)	1.79 ± 1.65
Medication:	
Prednisolone use at baseline	15 (36%)
Prednisolone dose (mg/day)	4.4 ± 1.2
DMARD use at baseline	21 (50%)
PPI	35 (83%)
SSRI	2 (5%)

BMI, body mass index; CRP, C-reactive protein; CT, computed tomography; dcSSc, diffuse cutaneous systemic sclerosis; DMARD, disease-modifying anti-rheumatic drug; DU, digital ulceration; ILD, interstitial lung disease; lcSSc, limited cutaneous systemic sclerosis; mRSS, modified Rodnan Skin Score; N, number; PAH, pulmonary arterial hypertension, PPI, proton pump inhibitor; SSRI, selective serotonin reuptake inhibitors; TSH, thyroid-stimulating hormone; Vit D3, 25-OH vitamin D.

### BMD

**Figure 1:**
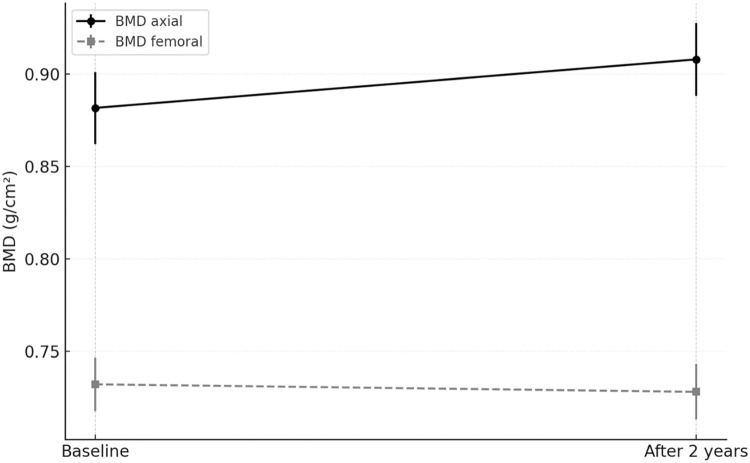
Changes of BMD in the total cohort over 2 years. BMD, bone mineral density.

Overall, antiresorptive treatment significantly improved axial BMD and corresponding T-score (*p* = .012, 95% CI [0.01, 0.05] and *p* = .028, 95% CI [0.03, 0.43]), while changes in femoral BMD were not statistically significant (Table 2 and Fig 1).

**Table 2:** Changes of BMD after 2 years

	Baseline	After 2 years	Δ	95% CI	*P* value
Total cohort (n = 42)					
BMD axial	0.88 ± 0.12	0.91 ± 0.12	0.03 ± 0.07	[0.01, 0.05]	**.012**
T-score axial	−2.50 ± 1.02	−2.27 ± 1.05	0.23 ± 0.63	[0.03, 0.43]	**.028**
BMD femoral	0.73 ± 0.09	0.73 ± 0.10	−0.00 ± 0.04	[−0.02, 0.01]	.500
T-score femoral	−2.24 ± 0.74	−2.28 ± 0.79	−0.04 ± 0.30	[−0.13, 0.06]	.440
Oral (n = 21)					
BMD axial	0.88 ± 0.15	0.90 ± 0.14	0.02 ± 0.06	[−0.01, 0.04]	.257
T-score axial	−2.55 ± 1.25	−2.45 ± 1.22	0.10 ± 0.54	[−0.16, 0.35]	.442
BMD femoral	0.75 ± 0.11	0.74 ± 0.12	−0.00 ± 0.05	[−0.03, 0.02]	.687
T-score femoral	−2.11 ± 0.89	−2.15 ± 0.98	−0.04 ± 0.38	[−0.22, 0.14]	.641
Parenteral (n = 21)					
BMD axial	0.88 ± 0.09	0.93 ± 0.10	0.05 ± 0.08	[0.01, 0.09]	**.026**
T-score axial	−2.46 ± 0.73	−2.09 ± 0.84	0.37 ± 0.69	[0.04, 0.70]	**.032**
BMD femoral	0.72 ± 0.07	0.71 ± 0.07	−0.00 ± 0.03	[−0.02, 0.01]	.539
T-score femoral	−2.37 ± 0.54	−2.41 ± 0.56	−0.03 ± 0.21	[−0.13, 0.06]	.481
Alendronate (n = 19)					
BMD axial	0.87 ± 0.15	0.89 ± 0.14	0.01 ± 0.06	[−0.02, 0.04]	.353
T-score axial	−2.59 ± 1.29	−2.46 ± 1.18	0.13 ± 0.50	[−0.11, 0.38]	.270
BMD femoral	0.74 ± 0.11	0.74 ± 0.11	−0.01 ± 0.05	[−0.03, 0.02]	.546
T-score femoral	−2.18 ± 0.86	−2.18 ± 0.90	−0.03 ± 0.37	[−0.22, 0.15]	.710
Zoledronate (n = 9)					
BMD axial	0.91 ± 0.10	0.94 ± 0.11	0.04 ± 0.09	[−0.03, 0.10]	.255
T-score axial	−2.23 ± 0.78	−1.97 ± 0.91	0.27 ± 0.71	[−0.28, 0.82]	.295
BMD femoral	0.70 ± 0.08	0.71 ± 0.08	0.00 ± 0.02	[−0.01, 0.02]	.714
T-score femoral	−2.46 ± 0.64	−2.44 ± 0.66	0.01 ± 0.17	[−0.12, 0.14]	.849
Denosumab (n = 11)					
BMD axial	0.85 ± 0.07	0.91 ± 0.10	0.06 ± 0.08	[0.00, 0.13]	**.049**
T-score axial	−2.77 ± 0.61	−2.24 ± 0.83	0.52 ± 0.70	[−0.02, 1.06]	.056
BMD femoral	0.72 ± 0.06	0.72 ± 0.07	−0.01 ± 0.03	[−0.03, 0.01]	.467
T-score femoral	−2.35 ± 0.48	−2.41 ± 0.50	−0.06 ± 0.25	[−0.23, 0.11]	.426

BMD, bone mineral density; n, number. Boldface values indicates statistically significant differences (p < 0.05).

Oral treatment: alendronate (n = 19) and risedronate (n = 2); parenteral treatment: zoledronate (n = 9), ibandronate (n = 1), and denosumab (n = 11).

**Figure 2:**
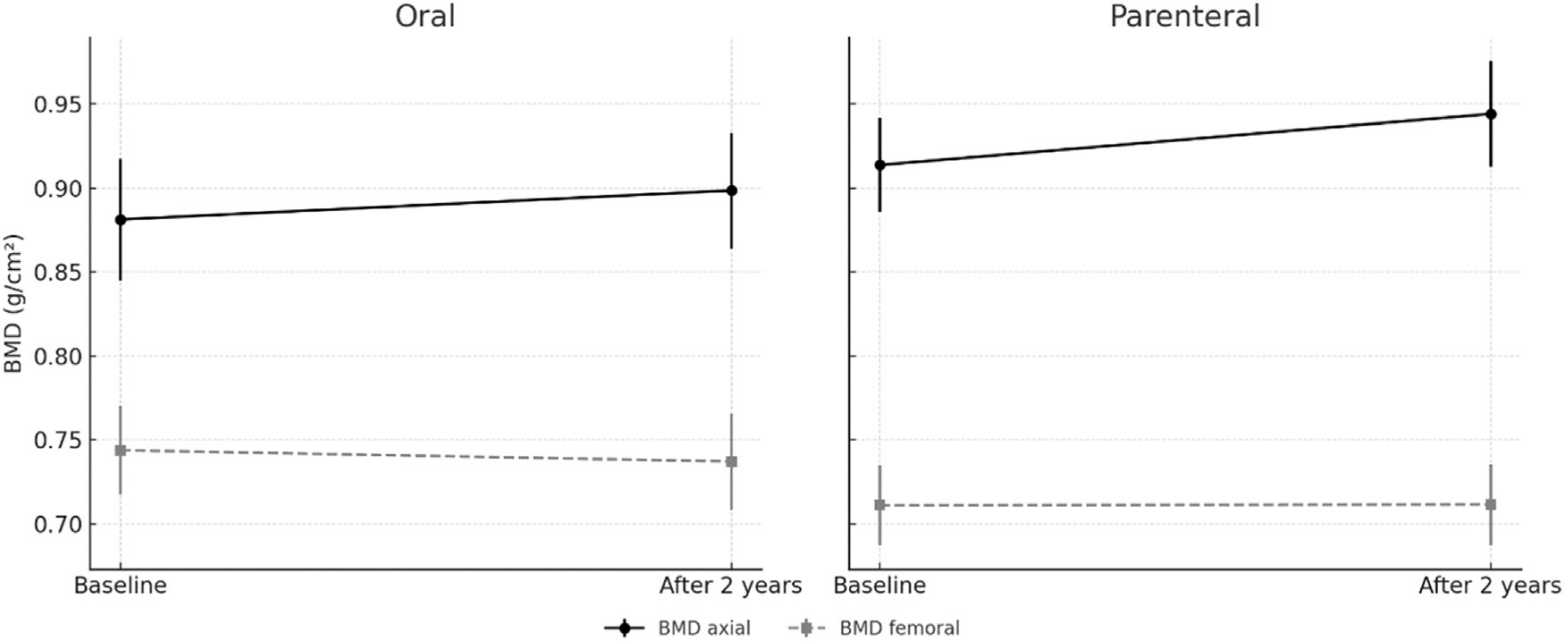
Changes in BMD with oral and parenteral antiresorptive treatment over 2 years in comparison. BMD, bone mineral density.

In further analysis, axial BMD and T-scores showed statistically significant improvements in patients treated with parenteral antiresorptive therapy (*p* = .026, 95% CI [0.01, 0.09] and *p* = 0.032, 95% CI [0.04, 0.70]), whereas no significant improvements of BMD were observed under oral therapy (Table 2 and Fig 2). However, these effects were not statistically significant when compared to each other (axial BMD and corresponding T-score *p* = .195 and *p* = .179; femoral BMD and corresponding T-score *p* = .949 and *p* = .945).

**Figure 3:**
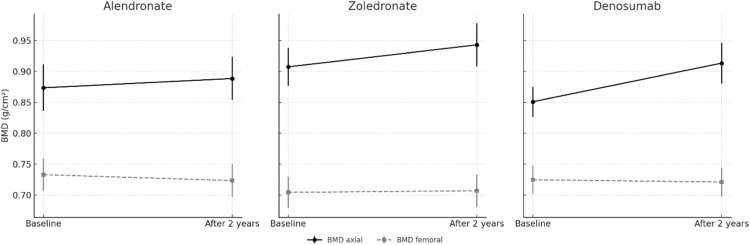
Changes in BMD with alendronate, zoledronate, and denosumab over 2 years in comparison. BMD, bone mineral density.

When analysing BMD changes under individual drugs, only patients treated with denosumab demonstrated a significant increase in axial BMD during therapy (*p* = .049, 95% CI [0.00, 0.13]) (Table 2 and Fig 3). However, the intergroup comparison using Kruskal–Wallis test showed no significant superiority of denosumab over the other drugs.

When considering the group undergoing oral bisphosphonate therapy, there was no significant difference in treatment response depending on oesophageal hypomotility or atony (BMD axial *p* = .992; BMD femoral *p* = .265).

In accordance with the DVO criteria for pharmacological treatment failure [18], 8 patients showed a drop in BMD >5% (5/19 patients with alendronate [26%], 1/9 patients with zoledronate [11%], 2/11 patients with denosumab [18%]) (Supplementary Table).

With regard to the response to antiresorptive therapy, multivariate regression analysis revealed no significant association with age, disease duration, BMI, SSc subtype, autoantibody profile, organ involvement, vitamin D status or supplementation, CRP, serum calcium levels, prednisolone use, proton pump inhibitors, or selective serotonin reuptake inhibitors. However, patients who met the criteria for formal treatment failure showed, on average, lower vitamin D levels (31.5 vs 36.5 ng/mL) and lower weekly supplementation doses (6825 vs 9082 IU), as well as higher CRP levels (1.21 vs 0.30 mg/dL) and higher daily prednisolone intake (2.50 vs 1.38 mg).

### Safety

No atypical (femoral) fractures as a potential side effect of (long-term) antiresorptive therapy were observed. One patient developed stage 1 ONJ according to the staging system of the American Association of Oral and Maxillofacial Surgeons during treatment with ibandronate [20]. The condition was managed conservatively with local antibacterial therapy. Fortunately, no surgical measures were necessary.

In 3/9 patients (33%), there was an acute-phase reaction after infusion of zoledronate. No patients experienced hypocalcaemia, acute renal failure, or new onset of atrial fibrillation. Three of 19 patients (16%) receiving oral bisphosphonates reported nonspecific gastrointestinal symptoms such as heartburn or epigastric discomfort. All these patients were diagnosed with oesophageal hypomotility or atony. Notably, no serious gastrointestinal adverse events such as ulcers or gastrointestinal bleeding occurred in our cohort.

## DISCUSSION

This retrospective study evaluated the efficacy and safety of antiresorptive drugs in patients with SSc. To date, no clinical trials have been published regarding the effectiveness and safety of specific osteoporosis treatments in this osteovulnerable patient population. The current medical literature contains only a single case report describing the use of denosumab in a patient with SSc, who showed an insufficient therapeutic response [21].

This study demonstrated that antiresorptive therapy is effective in patients with SSc. No osteoporotic fractures occurred in any patient during the course of treatment. BMD, assessed as a surrogate marker of therapeutic response [22,23] showed a statistically significant improvement in axial measurement across the entire cohort (+3.78%, *p* = .012), independent of demographic and clinical context factors. In contrast, no significant changes were observed in femoral BMD.

In further analysis comparing oral and parenteral administration of antiresorptive therapy, no statistically significant difference was observed between groups. However, only patients treated with parenteral therapy showed a significant improvement in axial BMD (+5.46%, *p* = 0.026), whereas patients treated with oral therapy did not. Interestingly, the treatment response in the oral administration group was not significantly different depending on the presence of oesophageal hypomotility or atony.

When analysing treatment effects, the greatest average increase in axial BMD was observed in patients treated with denosumab (+7.51%), followed by zoledronate (+4.19%), with the smallest gain seen in those receiving alendronate (+1.91%). Only denosumab was associated with a statistically significant increase in axial BMD during the treatment period (*p* = 0.049). Nevertheless, in the group-wise comparison, denosumab did not demonstrate a statistically significant advantage over the other antiresorptive drugs.

The distribution of axial BMD gains observed for the individual drugs in this study is consistent with previously published data for each drug, except for alendronate, which has typically been associated with an average increase of 3% to 5% over 2 years [[24], [25], [26]].

According to the DVO criteria, which define a loss of >5% in BMD over 2 years as a potential indicator of treatment failure [18], 8 patients in our cohort met this criterion. Most of these cases occurred under alendronate therapy (26%). Otherwise, it is important to note that, particularly with oral bisphosphonates, a lack of measurable increase in BMD does not necessarily indicate a reduced antifracture efficacy [18].

The observation that axial BMD increased more than femoral BMD is consistent with previous findings. Several studies have demonstrated that antiresorptive therapy results in more pronounced and faster increases in axial BMD when compared to the hip [23]. This difference is primarily attributable to the distinct bone composition at these sites, with the lumbar spine consisting largely of trabecular bone, which is more metabolically active and responds more quickly to changes in bone remodelling than the predominantly cortical bone of the femur [27]. However, the lack of a meaningful increase in femoral BMD across all treatment groups (including denosumab, for which an average increase of 4% to 5% at the femur would typically be expected [26]) suggests the influence of disease specific factors in SSc: It is well-established that trabecular bone has substantially greater vascularisation than the cortical bone [28]. Studies have demonstrated an association between bone quality and the extent of vascular damage [29]. Furthermore, peripheral quantitative computed tomography has shown that especially cortical bone mass is disproportionately reduced in patients with SSc compared with the general population [30]. Taken together, these points could explain the non-response of femoral BMD to treatment.

The overall tolerability of antiresorptive therapy in this cohort was good. Mild gastrointestinal symptoms occurred only in a small subset of patients receiving oral bisphosphonates, with an incidence as described in the normal population, and were not associated with serious complications. This observation is consistent with the findings of a meta-analysis involving 39,047 patients, which reported no increased risk of severe gastrointestinal complications in individuals treated with bisphosphonates compared with those in the control group [31].

However, one case of stage 1 ONJ was observed during treatment with ibandronate. Although ONJ remains a rare complication, its occurrence in the context of SSc warrants particular attention. Patients with SSc are known to have a higher prevalence of periodontal disease, and additional disease-related factors such as xerostomia, microstomia, and vascular compromise may further increase susceptibility [12,[32], [33], [34]]. Although the case observed in this study did not require surgical intervention and was managed conservatively, it underscores the need for thorough dental evaluation and appropriate preventive strategies before initiating antiresorptive therapy in this patient population.

No unexpected cardiovascular or renal adverse events occurred, and no signs of treatment-related hypocalcaemia were detected. Taken together, these findings support the safety of both oral and parenteral antiresorptive agents in patients with SSc when appropriately monitored.

It remains uncertain whether the prescribed therapies, particularly those administered orally, were consistently taken as directed by the patients. It is well-established that adherence to oral bisphosphonate therapy can be suboptimal in routine clinical practice, often due to gastrointestinal side effects [35]. Given the retrospective nature of the study, no biochemical markers of bone metabolism were available to objectively verify medication adherence independent of patient self-report [36]. Furthermore, gastrointestinal adverse events were not systematically recorded, for example, by using validated instruments such as the OPSAT-Q questionnaire [37]. As a result, the actual incidence of gastrointestinal adverse events may have been underestimated, potentially leading to premature discontinuation of therapy in some cases.

A further limitation of the study is the small sample size, which reflects the rarity of the underlying condition. SSc has a reported prevalence of 3.8 to 50 per 100,000, and the subset of patients with concomitant osteoporosis requiring treatment is accordingly limited [38].

Future studies should be conducted on a larger scale, ideally in a multicenter prospective setting, to evaluate the therapeutic response to osteoporosis-specific drugs in patients with SSc also with a special focus on femoral BMD. Such studies should also incorporate the systematic assessment of (gastrointestinal) adverse events and include bone metabolism markers to allow for a more comprehensive evaluation. Due to the poorer dental status and limited oral hygiene, it would be important to investigate the incidence of ONJ for patients with SSc in more detail.

In summary, antiresorptive therapies appear to be effective in patients with SSc. Subgroup analysis suggests that parenteral administration, particularly denosumab, may offer superior efficacy. Gastrointestinal adverse effects were mild and consistent with published rates in the general population, whereas ONJ could be more relevant than in the general population.

## CRediT authorship contribution statement

**Nils Schulz:** Writing – original draft, Visualization, Project administration, Methodology, Investigation, Formal analysis, Conceptualization. **Jonas Neumann:** Writing – review & editing, Visualization, Investigation. **Tim Wilhelmi:** Writing – review & editing, Formal analysis. **Pascal van Wijnen:** Writing – review & editing, Methodology. **Ulf Müller-Ladner:** Writing – review & editing, Supervision, Project administration. **Philipp Klemm:** Writing – review & editing, Project administration, Formal analysis, Conceptualization.

## Competing interests

All authors declare they have no competing interests.

## Appendix Supplementary materials

Image, application 1

## Footnotes

Supplementary material associated with this article can be found in the online version at doi:10.1016/j.ero.2025.05.008.
