# Supplementary material for: Effectiveness and safety of antiresorptive drugs in patients with systemic sclerosis—a retrospective monocentric study
Source: EULAR Rheumatol Open. 2025 Jun 26;1(3):81–8. doi: 10.1016/j.ero.2025.05.008 (PMC13292479; doi:10.1016/j.ero.2025.05.008)
Supplement: Supplementary file 1 [file mmc1.docx]

**Supplementary Table** Patients meeting DVO treatment failure criteria

| Patientnumber | Group | Δ BMD axial (%) | Δ BMD peripher (%) |
| --- | --- | --- | --- |
| 1 | Alendronate | -12.21 % | -6.33 % |
| 2 | Alendronate | -9.09 % | -5.26 % |
| 3 | Alendronate | -3.83 % | -15.83 % |
| 4 | Alendronate | +11.89 % | -10.35 % |
| 5 | Alendronate | +5.11 % | -6.31 % |
| 6 | Zoledronate | –6.48 % | -6.42 % |
| 7 | Denosumab | - | -6.59 % |
| 8 | Denosumab | 0 | -11.28 % |

BMD: bone mineral density
